# Variables associated with use of symptomatic medication during a headache attack in individuals with tension-type headache: a European study

**DOI:** 10.1186/s12883-020-1624-8

**Published:** 2020-02-01

**Authors:** César Fernández-de-las-Peñas, Maria Palacios-Ceña, Matteo Castaldo, Kelun Wang, Ángel Guerrero-Peral, Antonella Catena, Lars Arendt-Nielsen

**Affiliations:** 1 Department Physical Therapy, Occupational Therapy, Rehabilitation, and Physical Medicine, University Rey Juan Carlos, Alcorcón, Spain; 2 Department of Health Science and Technology, Faculty of Medicine, Center for Sensory-Motor Interaction (SMI), Aalborg University, Aalborg, Denmark; 3 Facultad de Ciencias de la Salud, Universidad Rey Juan Carlos, Avenida de Atenas s/n28922 Alcorcón, Madrid, Spain; 4 Master in Sport Physiotherapy, University of Siena, Siena, Italy; 5 Poliambulatorio Fisiocenter, Collecchio (Parma), Collecchio, Italy; 6 Headache Unit, Hospital Universitario de Valladolid, Valladolid, Spain

**Keywords:** Tension type headache, Medication intake, Pressure pain, Sensitization

## Abstract

**Background:**

Pharmacological treatment of patients with tension-type headache (TTH) includes symptomatic (acute) and prophylactic (preventive) medication. No previous study has investigated variables associated to symptomatic medication intake in TTH. Our aim was to assess the association of clinical, psychological and neurophysiological outcomes with the use and timing of the use of symptomatic medication in TTH.

**Methods:**

A longitudinal observational study was conducted. One hundred and sixty-eight (*n* = 168) patients with TTH participated. Pain features of the headache (intensity, frequency, duration), burden of headache (Headache Disability Inventory), sleep quality (Pittsburgh Sleep Quality Index), anxiety/depression (Hospital Anxiety and Depression Scale), trait/state anxiety levels (State-Trait Anxiety Inventory), and bilateral pressure pain thresholds on the temporalis, C5-C6 joint, second metacarpal and tibialis anterior were assessed. Symptomatic medication intake was also collected for a 6-months follow-up period. Differences between patients using or not using symptomatic medication, depending on self-perceived effectiveness, and time (early during an attack, i.e., the first 5 min, or when headache attack is intense) when the symptomatic medication was taken were calculated.

**Results:**

One hundred and thirty-six (*n* = 136, 80%) reported symptomatic medication intake for headache (73% NSAIDs). Sixteen (12%) reported no pain relief, 81 (59%) experienced moderate relief and 39 (29%) total pain relief. Fifty-eight (43%) took ‘early medication’ whereas 78 (57%) took ‘late medication’. Patients taking symptomatic medication in general showed lower headache frequency and lower depressive levels than those patients not taking medication. Symptomatic medication was more effective in patients with lower headache history, frequency, and duration, and lower emotional burden. No differences in pressure pain sensitivity were found depending on the self-perceived effectiveness of medication. Patients taking ‘late symptomatic’ medication exhibited more widespread pressure pain sensitivity than those taking ‘early medication’.

**Conclusions:**

This study found that the effectiveness of symptomatic medication was associated with better headache parameters (history, frequency, or duration) and lower emotional burden. Further, consuming early symptomatic medication at the beginning of a headache attack (the first 5 min) could limit widespread pressure pain sensitivity.

## Background

Tension-type headache (TTH) is a pain disorder showing a prevalence of 42% in the general population [1], an important socio-economic impact [2] and a relevant social and personal burden [3]. In the last Global Burden of Disease Study, headache was found to be the second most prevalent chronic pain condition in the world [4].

Pharmacological treatment of patients with TTH includes symptomatic (acute) and prophylactic medication. Symptomatic medication refers to the treatment of a single headache attack, whereas prophylactic medication refers to continued treatment used for preventing the headache attacks. Simple analgesics and non-steroidal anti-inflammatory drugs (NSAIDs) are the mainstays within the symptomatic therapy. The clinical practice guideline of European Federation of Neurological Societies (EFNS) recommends simple analgesics (i.e., paracetamol) and NSAIDs (i.e., ibuprofen or aspirin) as first-line for the symptomatic treatment of TTH, particularly the episodic form [5]. This recommendation agrees with results from some Cochrane reviews reporting moderate to high evidence for the use of paracetamol (1000 mg) [6] or ibuprofen (400 mg) [7], and low to moderate evidence for the use of ketoprofen (25 mg) [8] or aspirin (500 mg–1000 mg) [9], as effective acute medications for episodic TTH. A recent study observed that NSAIDs consumption in people with TTH almost reached 90% and that patient’s preferences on medication intake was slightly different from clinical guideline recommendations [10].

Although the mechanisms underlying TTH are not completely understood, current evidence supports the presence of hyper-excitability of the central nervous system as an important factor in the development of TTH pain [11]. Further, several associated factors including mood disorders (anxiety or depression) [12], sleep disturbances [13] or emotional stress [14] can also contribute to this excitable state. No study has previously investigated the association of these variables to symptomatic medication intake in TTH patients. Considering the impact [2] and personal burden [3] associated to this headache disorder, better understanding of the variables associated with symptomatic medication intake may help to better identify some critical areas for future research on symptomatic medication treatment of individuals with TTH. Therefore, the aim of this longitudinal observational study was to investigate potential associations of clinical, psychological and sensitivity outcomes with use of symptomatic medication in individuals suffering from TTH. We hypothesized that: 1, taking symptomatic medication would be associated with less frequent and debilitating headaches, lower anxiety and depressive levels and less widespread pressure sensitivity than not taking symptomatic medication; and, 2, taking symptomatic medication at the beginning of the headache attack would be associated with less frequent and debilitating headache, lower levels of anxiety and depressive levels and less widespread pressure pain sensitivity than taking symptomatic medication when the pain of the headache attack is intense.

## Methods

### Study design

The current analysis is included as part of a multicenter international headache study about medication intake in TTH. Some patients from the current study were also included in a previous study which data have been already previously published [15]. This previous analysis found that patients with TTH taking prophylactic medication (i.e., amitriptyline) had higher frequency and burden of headaches, worse sleep quality and higher depressive levels than those TTH patients not taking prophylactic medication [15]. In addition, prophylactic medication was less effective in those patients exhibiting generalized pressure pain sensitivity [15]. The current study presents new data, different outcomes, and different reasoning.

### Patient and public involvement statement

Patients were not involved in the development of the study design, development of research question or their recruitment. Results from the study are available to those participants who requested them.

### Participants

Patients with a diagnosis of TTH were recruited from three different university-based hospitals (University Rey Juan Carlos, Aalborg University, Parma University) from September 2014 to January 2017. Diagnosis was done according to the third edition of the International Classification of Headache Disorders, (ICHD3 beta, 2013) down to third-digit level (codes 2.2, 2.3) by a neurologist expert in headaches [16]. They were excluded if presented: 1, other primary and/or secondary headache; 2, medication overuse headache as defined by the ICHD-3 (beta); 3, history of neck or head trauma; 4, any systemic degenerative disease; 5, diagnosis of fibromyalgia syndrome; 6, received anesthetic blocks or botulinum toxin within the previous 6 months; 7, received physical treatment in the neck and head the previous 6 months; or, 8, pregnancy. All participants read and signed a consent form prior to their participation. The local Ethics Committee approved the study (URJC 23/2014, HUFA 14/104, Aalborg N20140063, CESU 5/2015).

### Headache diary

A headache diary for 4 weeks was used to substantiate the diagnosis and to record the clinical features at baseline [17]. On the diary, patients registered the frequency of headaches (days/week), the headache intensity on an 11-points numerical pain rate scale [18] (NPRS; 0: no pain, 10: the maximum pain), and the duration of the headache attack (hours/day). Patients monitored their medication intake in a diary for a 6 months period. Patients regularly taking symptomatic medication for at least 30% of their headache attacks during this period were considered as ‘taking medication intake’. In this diary, they registered if the symptomatic medication drug used for headache (when taken); the time when they take the medication: ‘early symptomatic’ (at the beginning of the attack, in the first 5 min) or ‘late symptomatic’ (when the headache was intense, defined as > 7 on a NPRS); and the self-perceived effectiveness of the medication (i.e., absent, moderate or total pain relief at 2 h without the use of other medication) [19]. Absent relief was defined as no effect or a reduction of less than 20% in the intensity of the headache; moderate pain relief was defined as a reduction of 50% of the intensity of the headache; whereas total pain relief was defined as a reduction of more than 80% of the headache intensity.

### Anxiety and depressive symptoms

The Hospital Anxiety and Depression Scale (HADS) is a 14-items self-report screening scale indicating the presence of anxiety and depressive symptom [20]. It consists of 7 items for evaluating anxiety (HADS-A) and 7 for depression (HADS-D). Each item scores on a Likert scale (0–3) giving a maximum score of 21 points for each subscale [21]. The HADS has shown good validity and internal consistency for being used in subjects with headache [22]. Anxiety and depressive symptoms were assessed at baseline.

### Trait and state anxiety levels

The State-Trait Anxiety Inventory (STAI) is a 40-items self-report scale assessing state (items l-20, STAI-S) and trait (items 21–40, STAI-T) levels of anxiety [23, 24]. The STAI-S items assess relatively enduring symptom of anxiety whereas the STAI-T items measures a stable propensity to experience anxiety, and tendencies to perceive stressful situations as threatening. Both subscales have shown good internal consistency scores [25]. In both scales, higher scores indicate greater state or trait anxiety. State and trait levels of anxiety were assessed at baseline.

### Burden of headache

The Headache Disability Inventory (HDI) was used to evaluate headache burden. This questionnaire uses 25-items that inquire about the perceived impact of headache on emotional functioning and daily life activities [26]. Thirteen items assess emotional burden (HDI-E, maximum score: 52) and 12 items assess physical burden (HDI-P, maximum score: 48). A greater score indicates a higher burden of headache. The HDI exhibits good stability in patients with headache [27]. The HDI was assessed at baseline.

### Sleep quality

The Pittsburgh Sleep Quality Index (PSQI) was used to assess sleep quality over the previous month by including 19 self-rated questions and 5 questions answered by bed- or room- mates [28]. The total score ranges from 0 to 21 where higher score indicates worse quality of sleep. This questionnaire has good internal consistency and test-retest reliability [29, 30]. Sleep quality was assessed at baseline.

### Pressure pain sensitivity

An electronic pressure algometer (Somedic® Algometer, Sollentuna, Sweden) was used to bilaterally assess pressure pain thresholds (PPT) over the temporalis muscle, the C5-C6 joint, the second metacarpal and the tibialis anterior muscle. PPT is defined as the minimal amount of pressure where a sense of pressure changes to pain. Pressure was increased at a rate of approximately 30 kPa/s. The mean of 3 trials on each point, with a 30s resting period for avoiding temporal summation of pain [31] was calculated and used for the main analyses. The order of assessment was randomized between subjects and the assessor was blinded to other outcomes. Participants practiced first on the wrist extensors of the right forearm. The reliability of pressure algometry has been found to be high [32]. Pressure pain sensitivity was assessed at baseline.

### Statistical analysis

Means and confidence intervals were calculated to describe the outcomes. The Kolmogorov-Smirnov test revealed that all quantitative data had a normal distribution (*P* > .05). Patients were grouped by use or not use of symptomatic medication, by those taking the medication as ‘early symptomatic’ or as ‘late symptomatic’, and by the self-rated effectiveness of the medication (absent, moderate or total pain-relief). Differences between grouped patients in clinical features, burden of headache (HDI-E, HDI-P), depression (HADS-D), anxiety (HADS-A, STAI-T, STAI-S) and sleep quality (PSQI) were compared using one-way analysis of variance (ANOVA). Also, a 2-way ANOVA was used to evaluate the differences in PPTs with side as within-subjects factor and group as between-subjects factor. The normality and homogeneity criteria were checked for the dependent variables with Kurtosis and Skewness for the normality and Levene’s test for the homogeneity criteria. Separate ANOVAs were performed for each variable. Regression analysis were conducted to determine that the differences observed with the ANOVA remained significant after the inclusion of multiple comparisons. As multiple comparisons were conducted in the main analysis, a Bonferroni-corrected alpha level of 0.025 (2 independent-samples t-tests) was required accept statistically significance.

## Results

### Clinical data of the sample

A total of 220 individuals with headache were screened for possible eligibility criteria. Finally, 170 patients (73% women) satisfied all the eligibility criteria, agreed to participate and signed the written informed consent. Fifty (*n* = 50) were excluded for: co-morbid migraine (*n* = 25), previous whiplash injury (*n* = 10), medication overuse headache (*n* = 10) and fibromyalgia (*n* = 5). One hundred and sixty-eight (*n* = 168, 99%) were finally included in the analysis as they returned the headache diary with medication intake data. One hundred and sixty (*n* = 160, 95%) were diagnosed with TTH associated to pericranial tenderness whereas the remaining 8 (5%) were not associated to pericranial tenderness. Demographic data and outcome measure scores are listed in Table 1.

**Table 1:** Clinical features, psychological and related-disability outcomes in patients with tension-type headache in the total sample and by symptomatic medication intake (*n* = 168)

	Total sample (*n* = 168)	Taking medication (*n* = 136)	No taking medication (*n* = 32)
Clinical Pain Features			
Gender (male/female) n (%)	45 (27%) / 123 (73%)	36 (27%) / 100 (73%)	9 (31%) / 23 (72%)
FETTH / CTTH n (%)^a^	94 (56%) / 74 (44%)	82 (60%) / 54 (40%)	12 (38%) / 20 (62%)
Age (years)	46 (44, 48)	45 (42, 48)	48 (43, 53)
Headache history (years)	10.5 (8.9, 12.1)	10.8 (8.8, 12.8)	10.2 (7.1, 13.3)
Headache intensity (0–10)	6.1 (5.7, 6.5)	6.0 (5.4, 6.6)	6.6 (5.8, 7.4)
Headache frequency (days/month) ^a^	16.7 (12.5, 20.9)	16.2 (14.0, 18.4)	19.3 (17.8, 20.8)
Headache duration (hours per attack)	7.3 (6.6, 8.0)	7.2 (6.4, 8.0)	7.6 (6.0, 9.2)
Prophylactic medication n (%)	40 (25%)	16 (12%)	24 (75%)
Psychological and disability-related outcomes			
HADS-D (0–21) ^a^	8.4 (7.7, 9.1)	7.5 (6.7, 8.3)	9.5 (8.0, 11.0)
HADS-A (0–21)	10.7 (9.7, 11.7)	10.7 (9.8, 11.6)	10.6 (9.0, 12.2)
HDI-P (0–48)	23.0 (21.8, 24.2)	22.5 (20.2, 24.8)	23.2 (18,6, 27.8)
HDI-E (0–52)	19.7 (17.5, 21.9)	18.0 (15.5, 20.5)	22.2 (16.5, 27.9)
STAI-T (0–60)	23.9 (22.7, 25.1)	23.8 (22.4, 25.2)	24.2 (21.3, 27.3)
STAI-S (0–60)	21.8 (20.8, 22.8)	21.6 (20.5, 22.7)	22.2 (20.3, 24.1)
PSQI (0–21)	8.0 (7.2, 8.8.)	8.2 (7.5, 8.9)	7.8 (5.8, 9.8)

Values are expressed as means (95% confidence interval); ^a^ Significant differences between groups (ANOVA, *P* < 0.025)

*FETTH* Frequent episodic tension type headache; *CTTH* Chronic tension type headache; *HADS* Hospital Anxiety and Depression Scale (*D* Depression; *A* Anxiety), *HDI* Headache Disability Inventory (*P* Physical; *E* Emotional), *STAI* State-Trait Anxiety Inventory (*T* Trait; *S* State); *PSQI* Pittsburgh Sleep Quality Index

### Taking symptomatic or not taking symptomatic medication

One hundred and thirty-six (81%) reported taking symptomatic medication for their headache: 62 (45.5%) took simple analgesics (paracetamol) whereas the remaining 74 (54.5%) took NSAID (ibuprofen: *n* = 57, 42%; ketoprofen: *n* = 7, 5%; naproxen: *n* = 10, 7.5%). Patients reported that they took symptomatic medication in 70% of the headache attacks (mean 8.8 ± 1 headache per month), not in all.

Significant differences in the distribution of individuals with frequent episodic (FETTH) and chronic (CTTH) tension-type headache (*P* = 0.021), headache frequency (*P* = 0.015) and depression (HADS-D, *P* = 0.021) were observed between patients taking or not taking symptomatic medication. Post hoc analysis revealed a higher proportion of FETTH patients, those with lower frequency of headaches, or lower depressive levels within the symptomatic medication intake group (Table 1). The regression analysis also showed the same significant differences between those individuals taking or not taking symptomatic medication (Additional file 1: Table S1). No differences in gender (*P* = 0.63), age (*P* = 0.26), headache history (*P* = 0.8), headache intensity (*P* = 0.3), headache duration (*P* = 0.6), HADS-A (*P* = 0.345), STAI-T (*P* = 0.739), STAI-S (*P* = 0.591), HDI-P (*P* = 0.791), HDI-E (*P* = 0.117) and PSQI (*P* = 0.631) were found between patients taking or not taking symptomatic medication (Table 1). Similarly, no significant differences in sensitivity to pressure pain were either reported (C5-C6: F = 0.882, *P* = 0.349; temporalis muscle: F = 0.109, *P* = 0.742), second metacarpal (F = 0.455, *P* = 0.501), or tibialis anterior muscle (F = 1.212, *P* = 0.385) (Table 2).

**Table 2:** Pressure pain thresholds (PPT, kPa) between individuals with tension-type headache by symptomatic medication intake (*n* = 168)

	Temporalis muscle	C5-C6 zygapophyseal joint	Second metacarpal	Tibialis anterior muscle
Taking medication (*n* = 136)				
Right side	210.4 (193.5, 227.3)	204.8 (185.0, 224.6)	248.3 (231.0, 265.6)	402.8 (368.1, 437.5)
Left side	191.9 (177.2, 206.6)	197.5 (180.0, 215.0)	240.0 (223.0, 257.0)	394.1 (360.0, 428.2)
No taking medication (*n* = 32)				
Right side	220.8 (192.8, 248.8)	209.9 (179.8, 240.0)	272.7 (237.0, 308.5)	428.8 (367.2, 490.4)
Left side	199.7 (167.8, 231.6)	195.5 (162.0, 229.0)	260.3 (227.0, 293.6)	434.8 (368.0, 501.6)

Values are expressed as means (95% confidence interval)

### Self-reported effectiveness of symptomatic treatment

Sixteen (12%) patients reported no pain relief, other 81 (59%) get moderate pain relief, whereas 39 (29%) experienced total pain relief with acute medication. Significant differences in the distribution of patients with FETTH and CTTH (*P* = 0.023), headache history (F = 4.317, *P* = 0.015), headache duration (F = 7.578, *P* = 0.001), headache frequency (F = 9.627, *P* < 0.001) or emotional burden of headache (HDI-E: F = 4.945, *P* = 0.009) were observed depending on the self-rated effectiveness of symptomatic medication: a higher proportion of FETTH patients, those with a shorter headache history, shorter duration of headache attacks, lower headache frequency or lower emotional burden of the headache reported total pain relief with symptomatic medication intake (Table 3). For regression analysis, patients were grouped as total pain relief or no total pain relief (no pain relief and moderate pain relief together). Again, the regression analysis revealed significant differences according to the effects of symptomatic medication (Additional file 1: Table S2).

**Table 3:** Clinical features, psychological and related-disability outcomes in patients with tension-type headache depending on the self-reported perception of pain relief with medication (*n* = 136)

	No Relief (*n* = 16)	Moderate Relief (*n* = 81)	Total Relief (*n* = 39)
Clinical Pain Features			
Gender (male/female) n (%)	5 (31%) / 11 (69%)	22 (27%) / 59 (73%)	9 (23%) / 30 (77%)
FETTH / CTTH n (%)^a^	10 (62%) / 6 (38%)	40 (49%) / 41 (51%)	32 (82%) / 7 (18%)
Age (years)	44 (40, 48)	47 (44, 52)	46 (42, 50)
Headache history (years) ^a^	13.5 (9.6, 17.4)	9.8 (7.5, 12.1)	4.2 (2.1, 6.3)
Headache intensity (0–10)	6.0 (5.5, 6.5)	6.2 (5.8, 6.6)	6.0 (5.5, 6.5)
Headache frequency (days/) ^a^	18.2 (13.5, 22.9)	19.0 (17.2, 20.8)	11.8 (8.4, 15.2)
Headache duration (hours per attack) ^a^	9.2 (7.2, 11.2)	8.1 (7.1, 9.1)	5.2 (4.0, 6.4)
Psychological and disability-related outcomes			
HADS-D (0–21)	8.9 (5.7, 12.1)	8.4 (7.4, 9.4)	7.7 (6.4, 9.0)
HADS-A (0–21)	9.8 (6.7, 12.9)	9.9 (8.9, 10.9)	11.0 (9.7, 12.3)
HDI-P (0–48)	24.0 (20.7, 27.3)	24.1 (21.2, 27.0)	21.1 (17.1, 25.1)
HDI-E (0–52) ^a^	26.6 (20.4, 32.8)	20.8 (17.8, 23.8)	14.7 (10.7, 18.7)
STAI-T (0–60)	23.1 (19.9, 26.3)	23.8 (22.3, 25.3)	24.4 (22.0, 26.8)
STAI-S (0–60)	20.8 (16.8, 24.8)	21.5 (20.4, 22.6)	22.4 (20.7, 24.1)
PSQI (0–21)	7.0 (5.2, 8.8)	8.8 (7.8, 9.8)	7.3 (6.1, 8.5)

Values are expressed as means (95% confidence interval);

^a^ Significant differences between the total relief group and no or moderate relief group (ANOVA, *P* < 0.025)

*FETTH* Frequent episodic tension type headache; *CTTH* Chronic tension type headache; *HADS* Hospital Anxiety and Depression Scale (*D* Depression; *A* Anxiety), *HDI* Headache Disability Inventory (*P* Physical; *E* Emotional), *STAI* State-Trait Anxiety Inventory (*T* Trait; *S* State); *PSQI* Pittsburgh Sleep Quality Index

No differences in gender (*P* = 0.673), age (*P* = 0.659), headache intensity (*P* = 0.609), HADS-A (*P* = 0.474), STAI-T (*P* = 0.800), STAI-S (*P* = 0.615), HDI-P (*P* = 0.467), PSQI (*P* = 0.155) and PPTs (C5-C6: F = 0.313, *P* = 0.732; temporalis muscle: F = 0.416, *P* = 0.661; second metacarpal: F = 1.460, *P* = 0.236, or tibialis anterior muscle: F = 0.110, *P* = 0.896) were found depending on the self-reported effectiveness of the symptomatic medication (Tables 3 and 4).

**Table 4:** Pressure pain thresholds (PPT, kPa) in individuals with tension-type headache depending on the self-reported perception of pain relief with medication (*n* = 136)

	Temporalis muscle	C5-C6 zygapophyseal joint	Second metacarpal	Tibialis anterior muscle
No Relief (*n* = 16)				
Right side	220.6 (201.4, 239.8)	221.9 (191.9, 251.9)	288.0 (264.1, 311.9)	415.7 (381.5, 449.9)
Left side	215.4 (194.4, 236.4)	215.6 (182.1, 249.1)	284.1 (254.4, 313.8)	424.9 (395.4, 454.4)
Moderate Relief (*n* = 81)				
Right side	215.7 (197.4, 234.0)	208.9 (184.8, 233.0)	253.6 (234.5, 272.7)	416.5 (377.2, 455.8)
Left side	194.8 (177.8, 211.8)	205.6 (183.8, 227.4)	243.4 (224.4, 262.4)	400.6 (364.4, 436.8)
Total Relief (*n* = 39)				
Right side	217.1 (190.8, 243.4)	198.2 (175.2, 221.3)	238.9 (210.2, 267.6)	417.8 (379.8, 455.8)
Left side	198.3 (179.4, 217.2)	193.4 (171.9, 214.9)	231.9 (210.2, 253.6)	404.7 (367.6, 441.8)

Values are expressed as means (95% confidence interval)

### Taking ‘early symptomatic’ or ‘late symptomatic’ medication

Fifty-eight (43%) reported taking ‘early symptomatic’ medication, whereas the remaining 78 (57%) took ‘late symptomatic’ medication. No differences were observed for any clinical or psychological outcome (FETTH/CTTH: *P* = 0.359; gender: *P* = 0.323; age: *P* = 0.545; headache history: *P* = 0.181; headache intensity: *P* = 0.914: headache duration: *P* = 0.68; headache frequency: *P* = 0.160; HADS-D: P0.443; HADS-A: *P* = 0.930; STAI-T: *P* = 0.675; STAI-S: *P* = 0.627; HDI-P: *P* = 0.366; HDI-E: *P* = 0.523; PSQI: *P* = 0.33) between patients depending on the time of the medication intake (Table 5). Significant differences in pressure pain sensitivity over the temporalis (F = 6.243, *P* = 0.014), second metacarpal (F = 5.731, *P* = 0.018) and tibialis anterior (F = 5.667, *P* = 0.019), but not over C5-C6 joint (F = 4.803, *P* = 0.030) were observed: individuals taking ‘late symptomatic’ medication exhibited more widespread pressure pain hypersensitivity than those patients taking ‘early symptomatic’ medication (Table 6). The regression analysis confirmed the association of lower PPTs (higher pressure pain sensitivity) in those patients taking ‘late symptomatic’ medication (Additional file 1: Table S3).

**Table 5:** Clinical features, psychological and related-disability outcomes in patients with tension-type headache depending on the time when the symptomatic medication intake (*n* = 136)

	Early medication (*n* = 58)	Late medication (*n* = 78)
Clinical Pain Features		
Gender (male/female) n (%)	16 (27%) / 42 (73%)	20 (26%) / 58 (74%)
FETTH / CTTH n (%)	38 (65%) / 20 (35%)	44 (56%) / 34 (46%)
Age (years)	45 (42, 48)	45 (41, 49)
Headache history (years)	12.5 (9.0, 15.0)	9.8 (7.4, 12.2)
Headache intensity (0–10)	6.0 (5.5, 6.5)	6.0 (5.6, 6.4)
Headache frequency (days/)	14.9 (12.6, 17.2)	17.0 (15.2, 18.8)
Headache duration (hours per attack)	7.4 (6.2, 8.6)	7.1 (6.1, 8.1)
Psychological and disability-related outcomes		
HADS-D (0–21)	7.1 (6.0, 8.2)	7.7 (7.0, 8.4)
HADS-A (0–21)	10.6 (9.2, 11.0)	10.8 (10.0, 11.6)
HDI-P (0–48)	23.9 (20.0, 27.8)	22.0 (19.7, 24.3)
HDI-E (0–52)	18.9 (15.3, 22.5)	17.4 (14.6, 20.2)
STAI-T (0–60)	23.4 (21.3, 25.5)	24.0 (22.3, 25.7)
STAI-S (0–60)	21.2 (19.8, 22.6)	21.7 (20.3, 23.1)
PSQI (0–21)	7.7 (6.5, 8.9)	8.5 (7.4, 9.6)

Values are expressed as means (95% confidence interval)

*FETTH* Frequent episodic tension type headache; *CTTH* Chronic tension type headache; *HADS* Hospital Anxiety and Depression Scale (*D* Depression; *A* Anxiety), *HDI* Headache Disability Inventory (*P* Physical; *E* Emotional), *STAI* State-Trait Anxiety Inventory (*T* Trait; *S* State); *PSQI* Pittsburgh Sleep Quality Index

**Table 6:** Differences in pressure pain thresholds (PPT, kPa) in individuals with tension-type headache depending on the time when the symptomatic medication intake (*n* = 136)

	Temporalis muscle^a^	C5-C6 zygapophyseal joint	Second metacarpal^a^	Tibialis anterior muscle ^a^
Early medication (*n* = 58)				
Right side	231.5 (201.4, 261.6)	228.4 (192.3, 264.5)	268.7 (237.5, 299.9)	449.7 (385.5, 513.9)
Left side	202.4 (176.7, 228.1)	219.9 (186.4, 253.4)	257.6 (227.6, 287.6)	441.7 (376.8, 506.6)
Late medication (*n* = 78)				
Right side	194.5 (176.2, 212.8)	185.4 (167.5, 203.3)	232.8 (212.3, 253.3)	358.2 (322.0, 394.4)
Left side	183.0 (167.0, 199.0)	180.5 (162.8, 198.2)	227.3 (207.3, 247.3)	359.5 (324.3, 394.7)

Values are expressed as means (95% confidence interval)

^a^ Significant differences between both groups (2-two way ANOVA test, *P* < 0.025)

## Discussion

This is the first study investigating variables associated with the use of symptomatic medication intake in patients with TTH and when the medication is taken (early or late during the headache attack). The use of symptomatic medication was associated with a lower headache frequency and lower depressive symptoms, but not to other clinical or psychological outcomes, in TTH. Patients with lower frequency and shorter duration of the headaches, shorter headache history, and lower emotional headache burden self-experienced better effects of symptomatic medication. Individuals taking symptomatic medication at the beginning of the headache (‘early symptomatic medication’) presented lower widespread pressure pain sensitivity than those taking the medication when the headache is intense (‘late symptomatic medication’).

### Symptomatic medication in TTH

In our study, 81% of our sample of patients with TTH reported taking medication for their headache attacks, data similar to a recent study conducted in Italy where 90% of individuals with TTH were symptomatic medication users [10]. In the current study, 45.5% of the patients took simple analgesics (i.e., paracetamol) and 54.5% took NSAID particularly ibuprofen. These data are similar to those previously reported by Affaitati et al. [10] and also agree with the recommendations from international guidelines [5].

Individuals with lower frequency of headaches, that is, those with more episodic attacks, tend to use symptomatic medication in a greater proportion than those with CTTH. Our data agree with a previous study reporting that chronic headache sufferers are more likely to use opioid-combination analgesics, but less likely to use paracetamol, aspirin or ibuprofen, than episodic headache subjects [33]. It seems that medication intake patterns may be different between patients with chronic or episodic headaches.

Interestingly, no differences in anxiety levels, headache burden, sleep quality or widespread pressure pain sensitivity were observed depending on the use or not use of symptomatic medication. However, we found that individuals taking acute medication showed lower depressive levels than those not taking medication. This finding may be related to the fact that depression is associated with headache frequency; therefore, individuals with lower headache frequency tend to exhibit lower depressive levels [34].

### Self-perceived effectiveness of symptomatic medication intake

In our study, 30% of patients with TTH taking symptomatic medication reported total pain relief, which agree with current data on effectiveness of acute medication [6–9]. Symptomatic medication was more effective in those patients with lower frequency and shorter duration of headaches, shorter history of pain, and lower emotional burden of headache. It seems that higher frequency of headache [35] and emotional factors [36] can lead to hyperalgesic response to the central nervous system; therefore, symptomatic medication may be more effective in patients with lower central sensitization. However, we did not observe differences in widespread pressure pain sensitivity, a manifestation of central sensitization, based on self-perceived effects of symptomatic medication. It is possible that several pain mechanisms are involved in the effectiveness of symptomatic medication in patients with TTH.

### Early or late taking symptomatic medication during the attack

An important finding of this study was that patients taking symptomatic medication at the beginning of the headache attack (‘early symptomatic medication’) showed lower widespread pressure pain sensitivity than those subjects taking the medication when the headache was intense (‘late symptomatic medication’). The presence of widespread sensitivity to pressure pain is a manifestation of central sensitization [11]. It has been suggested that long-lasting nociception are responsible for sensitization of the central nervous system and/or impaired supra-spinal modulation of the incoming stimuli, and the development of central sensitization [11]. It is possible that taking the symptomatic medication at the beginning of the headache attack (i.e., during the first 5 min) could prevent central sensitization by decreasing peripheral nociception, lessening the barrage to the central nervous system. This hypothesis agrees with the underlying mechanisms of symptomatic medication since either NSAID or paracetamol inhibits production of prostaglandins [37]. Since prostaglandins play a role in nociceptive process, early intake of symptomatic medication may reduce nociceptive barrage to the central nervous system. Further, this mechanism may be not associated with hypoalgesic effects induced by symptomatic medication, since widespread pressure sensitivity was not associated to self-perceived effectiveness of symptomatic medication. Future studies investigating the potential underlying mechanisms of clinical and neurophysiological changes related to medication intake are needed. Additionally, current results open future clinical trials for investigating the effects of symptomatic medication parameters analyzed in this study in central sensitization in people presenting with TTH. Finally, patients should be educated to an ‘early’ symptomatic medication intake in clinical practice for decreasing potential central sensitization.

### Strength and limitations

Although strengths of the current study include a large sample size, the inclusion of patients accordingly to restricted diagnostic criteria and the use of diagnostic diary some limitations should be also recognized. First, although the sample size was large, a sample size calculation was not possible; therefore, it is possible that some comparisons could have been underestimated. Second, we included patients with TTH from tertiary headache centers; therefore, results may be not representative of the general population. Nevertheless, prevalence of symptomatic medication intake observed in our study was similar to previous population-based studies. In addition, subjects suffering from TTH seen in a tertiary center usually also exhibit coexisting migraine symptoms; although diagnosis of concomitant primary headaches was carefully excluded. Another potential limitation in relation to our data on symptomatic medication intake was the time period for consider as ‘early symptomatic medication’ (5 min) which can be slightly restricted. Third, data for depression and sleep quality were smaller than expected, which could be related to the questionnaires used in the study. For instance, the HADS is considered a screening rather than a diagnostic instrument for depressive symptoms with a tendency to underestimate its prevalence [38]. We do not know if the use of other instruments could lead to different results. In fact, it should be considered that most outcomes were self-reported and their psychometric data in the languages involved ion this study, e.g., Dutch, Spanish, Italian, have not been completely determined. Finally, we do not know if the identified associations will maintain with longer follow-ups since symptomatic medication intake patterns could change with time in TTH.

## Conclusions

This study found that the use of symptomatic acute medication for TTH was associated with a lower headache frequency and lower depressive symptoms, but not to other clinical/psychological outcomes. Higher effectiveness of symptomatic medication was associated with lower frequency and shorter duration of the headaches, shorter headache history, and lower emotional headache burden. Finally, early intake of the symptomatic medication, i.e., at the beginning of the headache attack, was associated with lower widespread pressure pain hypersensitivity.

## Supplementary information

**Additional file 1: Table S1.** Regression analysis depending on taking (grouped as 0) or not taking (grouped as 1) medication. **Table S2.** Regression analysis depending on the effects of medication intake (no pain relief grouped as 0; total pain relief grouped as 1). **Table S3.** Regression analysis depending on taking ‘early’ (grouped as 0) or ‘late’ (grouped as 1) symptomatic medication.

## Data Availability

The datasets used and/or analyzed during the current study are available from the corresponding author only on reasonable request.

## Abbreviations

ANOVA: Analysis of Variance
HADS: Hospital Anxiety and Depression Scale
HDI: Headache Disability Inventory
ICHD3: International Classification of Headache Disorders, third edition
NPRS: Numerical Pain Rate Scale
NSAIDs: Non-Steroidal Anti-Inflammatory Drugs
PPT: Pressure Pain Thresholds
STAI: State-Trait Anxiety Inventory
TTH: Tension-Type Headache
