# Supplementary material for: Variables associated with use of symptomatic medication during a headache attack in individuals with tension-type headache: a European study
Source: BMC Neurol. 2020 Feb 1;20:43. doi: 10.1186/s12883-020-1624-8 (PMC6995039; doi:10.1186/s12883-020-1624-8)
Supplement: Supplementary file 1 — Additional file 1: Table S1. Regression analysis depending on taking (grouped as 0) or not taking (grouped as 1) medication. Table S2. Regression analysis depending on the effects of medication intake (no pain relief grouped as 0; total pain relief grouped as 1). Table S3. Regression analysis depending on taking ‘early’ (grouped as 0) or ‘late’ (grouped as 1) symptomatic medication. [file 12883_2020_1624_MOESM1_ESM.docx]

**Supplementary Table 1**: Regression analysis depending on taking (grouped as 0) or not taking (grouped as 1) medication

| Outcomes | Odd Ratio (95% Confidence Interval) | P value |
| --- | --- | --- |
| Headache Frequency | 1.25 (95% CI 1.04, 1.46) | 0.015 |
| HADS-D | 1.14 (95% CI 1.05, 1.23) | 0.025 |

**Supplementary Table 2**: Regression analysis depending on the effects of medication intake ( no pain relief grouped as 0; total pain relief grouped as 1)

| Outcomes | Odd Ratio (95% Confidence Interval) | P value |
| --- | --- | --- |
| Headache Frequency | 0.95 (95% CI 0.92, 0.98) | 0.020 |
| Headache Duration | 0.77 (95% CI 0.62, 0.92) | 0.014 |
| Headache History | 0.97 (95% CI 0.96, 0.98) | 0.024 |
| HDI-E | 0.96 (95% CI 0.94, 0.98) | 0.021 |

**Supplementary Table 3**: Regression analysis depending on taking ‘early’ (grouped as 0) or ‘late’ (grouped as 1) symptomatic medication

| Outcomes | Odd Ratio (95% Confidence Interval) | P value |
| --- | --- | --- |
| PPT temporalis | 0.97 (95% CI 0.96, 0.98) | 0.025 |
| PPT second metacarpal | 0.98 (95% CI 0.97, 0.99) | 0.021 |
| PPT tibialis anterior | 0.98 (95% CI 0.97, 0.99) | 0.023 |
